# Burden of pneumococcal disease in adults aged 65 years and older: an Australian perspective

**DOI:** 10.1186/s41479-016-0008-8

**Published:** 2016-06-27

**Authors:** Kylie Earle, Scott Williams

**Affiliations:** 1 Pfizer Australia, Access and Public Affairs, Australia 38-42 Wharf Road, West Ryde, Sydney, 2114 NSW Australia; 2 Pfizer Australia, Vaccines, Sydney, Australia

**Keywords:** Pneumonia, Pneumococcal disease, Burden of disease, Adults, *Streptococcus pneumoniae*

## Abstract

**Background:**

The burden of pneumococcal disease in adults aged 65 years and older in Australia is not well defined. This retrospective cross-sectional study calculated rates for pneumococcal pneumonia using data from the Australian Institute of Health and Welfare and from the Bettering Evaluation and Care of Health program.

**Methods:**

Invasive pneumococcal disease (IPD) incidence was calculated using National Notifiable Diseases Surveillance System data. Population estimates and pneumonia mortality data were from the Australian Bureau of Statistics. Medical costs were derived from Australian Refined Diagnosis Related Groups and the literature. Clinical and economic burden of pneumococcal pneumonia hospitalisations and general practitioner (GP) visits were described and compared with IPD.

**Results:**

For adults aged ≥65 years, pneumococcal pneumonia hospitalisation incidence was 274 per 100,000 population in 2011–2012. From 2004 to 2012, a mean of 2235 pneumonia hospitalisation deaths were recorded, corresponding to a case fatality rate of 6.1 %. GP visits accounted for the largest portion of healthcare encounters, with an annual average of 455 pneumococcal pneumonia GP visits per 100,000 population from 2008 to 2013. In 2012, IPD incidence was 19 per 100,000 population. The estimated annual costs of treating pneumococcal pneumonia hospitalisations and GP visits were A$55,722,136 and A$1,604,189, respectively. Estimated costs for IPD were A$1,172,986.

**Conclusions:**

The healthcare and economic burden of pneumococcal disease in adults aged ≥65 years in Australia is substantial, with the incidence of pneumococcal pneumonia hospitalisation nearly 15-fold higher than for IPD. Despite this, it remains less recognised than other infectious diseases such as influenza.

## Background

*Streptococcus pneumoniae* is responsible for significant morbidity and mortality, especially in young children and the elderly [1]. In adults, the risk of contracting pneumococcal disease increases markedly with age [2, 3]. Currently, more than 3.3 million adults in Australia are aged 65 years and older, accounting for 14 % of the total population [4]. High rates of pneumonia occur among this population, which is also at an increased risk for invasive pneumococcal disease (IPD) [5].

As a nationally notifiable disease, IPD data have been collected in Australia since 2001. Consequently, a large quantity of data is available for evaluation of IPD disease burden in adults aged ≥65 years. However, IPD likely represents only a small portion of total pneumococcal disease, with non-invasive pneumococcal pneumonia expected to occur much more frequently.

Complications from pneumonia include long-term functional and cognitive impairment [6], and cardiovascular dysfunction [7, 8]. Approximately 12 to 20 % of patients admitted for pneumococcal pneumonia reported ≥1 associated cardiac event upon admission or during hospitalisation, with associated risk for higher mortality [7, 9]; *S. pneumoniae* was the most frequently identified pathogen among a cohort of hospitalised patients with pneumonia with cardiac events (41 % of patients) [8].

The aim of this retrospective study is to provide current estimates of the clinical and economic burden associated with pneumococcal pneumonia and IPD in individuals aged ≥65 years in Australia by analysing data available from multiple national databases.

## Methods

### Study design

Data from 4 national databases and previously published studies [5, 10, 11] were used to estimate the burden of pneumococcal disease. For the purposes of this study, pneumococcal disease was limited to pneumonia and IPD. Therefore, the study estimated the burden of hospitalisation for all-cause and pneumococcal pneumonia, the frequency of general practitioner (GP) visits for all-cause and pneumococcal pneumonia, and total IPD. The case fatality rates for all-cause pneumonia hospitalisation were also calculated. The costs associated with treating pneumonia and IPD in hospital and at GP visits were estimated. All persons aged ≥65 years living in Australia who had a recorded hospital or GP visit for pneumonia between 2001 and 2013 were included. Similarly, all patients aged ≥65 years who were diagnosed with IPD in 2012 were included. As this study examined anonymised data in national databases, ethics approval and informed consent were not required.

### Data collection and analysis

Data for all-cause pneumonia hospitalisation were obtained from the Australian Institute of Health and Welfare (AIHW) for financial years 2001–2012 using the *International Classification of Diseases, 10th Revision, Australian Modification* (ICD-10-AM) codes J12–J18. Data for all-cause pneumonia GP visits were provided by the University of Sydney from the Bettering Evaluation and Care of Health (BEACH) program from April 2008 to March 2013 [12]. Data for total IPD were extracted from the National Notifiable Diseases Surveillance System (NNDSS) IPD Public Data Set from 2012.

Incidences were calculated by dividing the number of pneumonia hospitalisations/IPD cases by the Australian population for the corresponding years, according to the Australian Bureau of Statistics (ABS) [13]. The frequency of pneumonia GP visits were calculated by dividing the number of GP visits by the average Australian population for the corresponding years from the ABS population projections [14]. Data from AIHW, BEACH, and NNDSS were stratified into age groups of 65–74 years, 75–84 years, and ≥85 years. The IPD data from the NNDSS were also stratified according to the clinical presentations of pneumonia, bacteraemia, and meningitis.

Calculations of the proportions of pneumococcal pneumonia in Australia were based on the midpoint between the Australian Community-Acquired Pneumonia Study (ACAPS) by Charles et al. (13.9 %) [10] and the systematic review by Said et al. (27.3 %) [5]. This approach was taken because the results in the ACAPS may be an underestimate, as more than half of the cases were recorded as ‘no aetiology’ (54.4 %) and a large proportion of pneumococcal CAP episodes were identified from positive Alere BinaxNOW® *Streptococcus pneumoniae* urinary antigen test (Alere Binax, United States) data alone (58 of 123). The sensitivity of BinaxNOW (Binax) has been estimated in recent systematic reviews and meta-analyses to be 66–75 % [15, 16]. A study of the diagnostic accuracy of a newly developed serotype-specific antigen test estimated that the addition of this test to conventional diagnostic methods increased the prevalence of *S. pneumoniae* community-acquired pneumonia by 39 % [17]. Other techniques such as quantitative PCR analysis followed by capsular sequence typing and inhibition multiplex immunoassay have also been identified, indicating that the true proportion of pneumococcal pneumonia may be higher. In one study, pneumococcus was detected in samples from 56 % more patients compared with conventional methods including Binax [18]. In contrast, the results from the systematic review may be an overestimate, as diagnosis of pneumococcal CAP cases was made by sputum culture only, without other microbiological test confirmation or newer diagnostic methods.

Data for all-cause pneumonia deaths were supplied from the ABS. Case fatality rates (CFRs) were calculated by dividing the average number of deaths attributed to ICD-10-AM codes J12–J18 from the 2004 to 2012 financial years by the average number of pneumonia hospitalisations (J12–J18) from the corresponding period.

Data to estimate the costs of treating pneumonia hospitalisations and IPD were derived from the National Hospital Cost Data Collection for public hospitals (Australian Refined Diagnosis Related Groups [AR-DRG]) [19]; private hospitals were excluded, as the cost weights do not include Medicare Benefits Schedule (MBS) costs, and therefore underestimate the total treatment cost. The AR-DRG items selected were E62A, E62B, and E62C for pneumonia hospitalisation; T60A and T60B for bacteraemia; and B72A and B72B for meningitis. Data to estimate the costs of treating pneumonia GP visits were sourced from Li and colleagues [11]. As Australian costs for pneumococcal pneumonia are not available, it was assumed that costs were the same as those associated with the treatment of all-cause pneumonia hospitalisation and GP visits. Primary costs per pneumonia episode for GP visits included ongoing care, specialists, medications, diagnostics, and allied health services (physiotherapy).

All authors had full access to all data in the study.

## Results

### Pneumonia hospitalisation

**Fig. 1:**
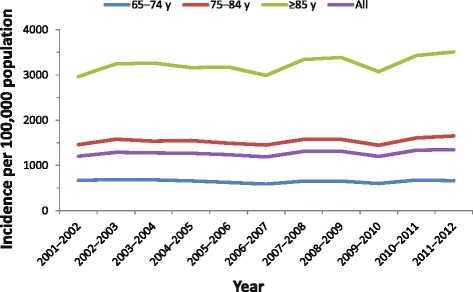
All-cause pneumonia hospitalisations for financial years 2001–2012. Incidence of all-cause pneumonia hospitalisations are shown per 100,000 population for adults aged 65–74 years, 75–84 years, ≥85 years, and all 3 age groups combined

Hospitalisation incidence for all-cause pneumonia in adults aged ≥65 years between 2001 and 2012 is shown in Fig. 1. For the financial year 2011–2012, 43,336 pneumonia hospitalisations were reported, corresponding to an incidence rate of 1347 per 100,000 population (Table 1). Incidence rates were higher in subjects ≥85 years of age (3507 per 100,000 population) compared with those 65–74 years old (662 per 100,000). Pneumococcal pneumonia accounted for 20.6 % (13.9–27.3 %) of hospitalisations, with an incidence of 274 per 100,000 population (Table 1).

**Table 1:** Annual Australian age-specific incidence of pneumococcal disease

	Age group, y			
	65–74	75–84	≥85	All
Australian population	1,789,563	1,028,247	438,797	3,256,607
*Pneumonia hospitalisations* ^*a*^				
Total number of pneumonia hospitalisations (all-cause)	11,779	16,818	14,739	43,336
Incidence of pneumonia hospitalisations (all-cause)^b^	658	1,636	3,359	1,331
Percent due to pneumococci^c^	20.6 %	20.6 %	20.6 %	20.6 %
Incidence of pneumococcal pneumonia hospitalisations	136	337	692	274
*Pneumonia GP visits* ^*d*^				
Total number of pneumonia GP visits (all-cause)	26,600	28,800	22,500	78,000
Frequency of pneumonia GP visits (all-cause)^b^	1,591	2,860	5,485	2,525
Percentage referred to hospital and/or ED^e^	12.5 %	12.5 %	12.5 %	12.5 %
Frequency of pneumonia GP visits (without hospitalisation)^b^	1,392	2,502	4,799	2,209
Percent due to pneumococci^c^	20.6 %	20.6 %	20.6 %	20.6 %
Frequency of pneumococcal pneumonia GP visits^b^	287	515	989	455
*IPD* ^*f*^				
Total number of IPD cases	233	216	164	613
Incidence				
IPD^b^	13	21	37	19
Pneumonia^b^	9	16	27	13
Bacteraemia^b^	2	3	6	3
Meningitis^b^	1	1	0	1

*Abbreviations*: *ED* emergency department, *GP* general practitioner, *IPD* invasive pneumococcal disease

^a^Pneumonia hospitalisation data from financial year 2011–12

^b^Per 100,000 population

^c^Calculated as the midpoint between the ACAPS study by Charles et al. (13.9 %) [10] and the systematic review by Said et al. (27.3 %) [5]

^d^Annual pneumonia GP visit data from April 2008 to March 2013

^e^BEACH calculated that 12.5 % of patients were referred to hospital and/or emergency departments [12]

^f^IPD data from 2012

### Pneumonia GP visits

Between April 2008 and March 2013, the average annual number of GP visits in Australia was reported as 119,300,000 visits. Of these, 78,000 were documented as pneumonia GP visits by adults aged ≥65 years, corresponding to approximately 2525 (95 % CI 2246–2800) visits per 100,000 population (Table 1). The frequency of GP visits for all-cause pneumonia ranged from 1591 per 100,000 in patients aged 65–74 years to 5485 per 100,000 in patients aged ≥85 years.

Because pneumonia GP visits are not specified by pathogen, application of the same proportion of all-cause pneumonia calculated as pneumococcal pneumonia (20.6 %) was used to estimate the frequency of GP visits for pneumococcal pneumonia at 455 per 100,000 for all adults 65 years and older. These values ranged from 287 to 989 per 100,000 in the younger and older age subgroups, respectively (Table 1).

### Invasive pneumococcal disease

In 2012, a total of 613 IPD cases were reported in adults aged ≥65 years in Australia, corresponding to an incidence of 19 cases per 100,000 population. Of these cases, 72 % were diagnosed as pneumonia (*n* = 439), 15 % as bacteraemia (*n* = 89 cases), 5 % as meningitis (*n* = 28 cases), and 9 % as other (*n* = 54 cases). A clinical presentation was not recorded for all cases of IPD (Table 1).

The estimate of invasive pneumonia is likely a significant underestimate as blood cultures are frequently not taken.

### Case fatality rates

In 2011, 118,553 deaths due to all causes in adults ≥65 years of age were reported to the AIHW. Between 2004 and 2012, the mean number of pneumonia hospitalisation deaths per year in adults aged ≥65 years attributed to ICD-10-AM codes J12–J18 was 2235.1, which corresponds to a CFR of 6.1 %. The 30-day fatality rate for pneumococcal pneumonia was estimated at 8.1 % based on the number of pneumococcal pneumonia deaths reported by Charles et al. [10] (PGP Charles, personal email communication, March 10, 2015). In this population, the CFR associated with IPD ranged from 14.3 to 16.4 % [20, 21].

### Economic burden of pneumonia

The total medical costs associated with public hospitalisation for pneumonia and the IPD clinical presentations of bacteraemia and meningitis were based on data from the Australian National Hospital Cost Data Collection. For AR-DRG items E62A, E62B, and E62C, pneumonia hospitalisation costs were A$6242 per person. For bacteraemia (AR-DRG items T60A and T60B), the average weighted treatment cost was A$10,148. The estimated average cost per pneumonia GP visit was based on the findings of Li and colleagues [11], which examined data collected from April 2006 to March 2009 by BEACH. An estimated A$114 was spent on 2 GP visits per episode of pneumonia. In 2012, the estimated annual cost of treating IPD was A$1,172,986.

Costs associated with all-cause pneumonia hospitalisation were estimated at A$270,495,804; costs associated with pneumococcal pneumonia hospitalisation were estimated at A$55,722,136. Estimated costs for GP visits for all-cause and pneumococcal pneumonia were A$8,899,800 and A$1,604,189, respectively. Taken together, the annual total cost of treating pneumococcal disease in adults aged ≥65 years was A$58,499,310.

## Discussion

**Fig. 2:**
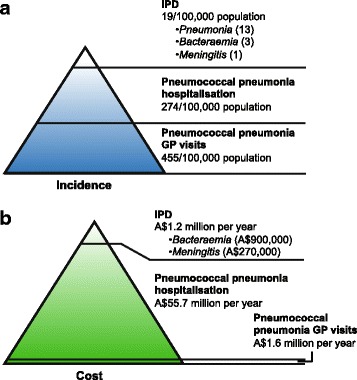
Distribution of pneumococcal disease (**a**) incidence and (**b**) associated costs. Estimated incidence per 100,000 population and costs associated with IPD, pneumococcal pneumonia hospitalisation, and pneumococcal pneumonia GP visits. Data for IPD are also shown by clinical presentation (pneumonia, bacteraemia and meningitis). GP, general practitioner; IPD, invasive pneumococcal disease

The clinical and economic burden of pneumococcal disease in adults aged 65 years and older in Australia is substantial, but has not been well described. IPD data have been collected in Australia since 2001, but relatively few publications have been produced despite robust datasets. Although most data available for pneumococcal disease relate to IPD, pneumococcal pneumonia represents a much larger but previously undescribed disease burden in elderly adults in Australia. We estimated that compared with IPD incidence, the rate of pneumococcal pneumonia hospitalisation and frequency of pneumococcal pneumonia GP visits were nearly 15- and 24-fold higher, respectively (Fig. 2a). These values climb markedly with age, reaching as high as 5780 GP visits per 100,000 population in adults aged 85 and older. To reduce the possibility of overcounting pneumonia GP visits, BEACH calculated that 12.5 % of patients were referred to hospital and/or emergency departments for pneumonia [12]. As a result, GP visit frequency fell slightly to 2209 per 100,000 population, but remained positively correlated with age.

Considering the frequency of healthcare encounters for pneumonia, the economic burden of treating this disease in adults aged 65 years and older is correspondingly large. Healthcare costs associated with pneumonia are significant: approximately €10–12 billion is spent annually to treat pneumonia patients in Europe [6, 22], and in the United States (US) approximately US$5–7 billion is spent on adults with pneumococcal disease [23, 24]. Costs are higher in adults ≥65 years of age when compared with ambulatory patients <65 years old [6, 25]. Based on data in this report, the majority of the financial burden of pneumococcal disease is for hospitalisation. More than A$50 million was spent on hospitalisation for pneumococcal pneumonia in 2011–2012, compared with approximately A$1.2 million that was spent on IPD cases in a similar period (Fig. 2b).

Strategies to reduce pneumococcal disease burden are primarily centred on vaccination. The 23-valent pneumococcal polysaccharide vaccine (PPSV23) is included in the Australian national immunisation programme for adults aged 65 years and over for protection against IPD, but protection wanes by 5 years after vaccination [26]. Although effective against IPD, a recent large-scale study of PPSV23 vaccination in more than 152,000 healthy adults clearly demonstrated no vaccine effect on pneumonia incidence [27]. These findings were confirmed in another study that showed vaccination was associated with a 42 % reduction in risk of IPD, but had no effect on pneumonia hospitalisation rates [28].

Although beneficial herd effects on all-cause pneumonia hospitalisation rates in older adults stemming from paediatric pneumococcal conjugate vaccine immunisation programmes have been observed in the US, these findings have not been replicated in Australia [29]. Hence, in some countries such as Australia, indirect effects from paediatric programs may foster improvements in disease burden for IPD in older adults, but may not affect the significant burden of pneumonia in these patients. These observations underscore the need for a mechanism to protect older adults from pneumococcal pneumonia that does not rely on herd effects. Results from a large European study of the 13-valent pneumococcal conjugate vaccine (PCV13) in adults aged ≥65 years indicate a vaccine effectiveness of 45.6 % in preventing the first episode of vaccine-serotype pneumococcal pneumonia and 75 % effectiveness against first episodes of vaccine-type IPD [30], which may have relevance for older adults in Australia. Indeed, a recent recommendation was made to include PCV13 on the national immunisation programme for the prevention of pneumococcal pneumonia and IPD in adults aged 65 years and over [31].

Given the presence of national immunisation programs for pneumococcal disease and influenza in older adults, comparison of the relative burdens of these illnesses may also be appropriate. Approximately 5 to 10 % of adults in Australia are also affected by respiratory infections caused by influenza, but these values are likely to be an underestimate of disease burden [32]. Despite an overall vaccine effectiveness of 50.9 % in elderly patients, influenza remains a substantial clinical and economic burden in Australia [32]. Between 2000 and 2006, more than 310,000 GP visits for influenza were recorded annually. The annual incidence of influenza hospitalisations in elderly adults ranged from 83.8 (95 % CI 55.7–111.9) per 100,000 population in individuals aged 65–74 years up to 378.2 (95 % CI 238.2–518.3) per 100,000 population in individuals >85 years of age [33]. Costs associated with influenza GP visits in adults aged 65–84 years were estimated at more than A$822,000 per year, with annual hospitalisation costs in those aged ≥65 years exceeding A$28.5 million. These costs are approximately half of the estimates for pneumococcal pneumonia in this study. Compared with pneumococcal pneumonia, the disease burden of influenza has been better studied and better recognised by healthcare professionals and the public. Influenza vaccination rates are also higher than those for pneumococcal vaccination [34], suggesting that a broader knowledge base to support public and medical professional awareness may contribute to higher vaccine uptake.

The current study adds much needed information surrounding the burden of pneumococcal pneumonia in elderly adults in Australia and may help in increasing awareness among healthcare professionals. The data presented provides essential information aimed at reducing the significant disease burden, such as the prevalence of pneumococcal pneumonia and the associated treatment costs.

A limitation of the present study stems from the majority of Australian pneumonia hospitalisations being reported as all-cause pneumonia; most cases are not categorised, and, therefore, the absolute proportion of pneumonia caused by *S. pneumoniae* remains somewhat uncertain. Less data on the aetiology of GP treated pneumonias are available than for hospitalised pneumonias, but there is a suggestion that pneumococcal pneumonia is more likely to be associated with increased severity and hence more likely to result in hospitalisation. For simplicity, the same calculations of the proportions of pneumococcal pneumonia for both pneumonia hospitalisation and pneumonia GP visits were undertaken, but this may result in an underestimate of pneumonia hospitalisation and an overestimate of pneumonia GP visits. In addition, diagnosis of hospitalised pneumonia is based on discharge coding and diagnosis of GP treated pneumonia is based on physician reporting. A recent paper suggested that a diagnosis of pneumonia was excluded for one-third of patients following a computer tomography (CT) scan [35].

Another limitation of the study is that the available data to inform this analysis were sourced from three different databases. Although there are overlaps with the time periods of data collection, it does mean that different populations were analysed. Despite these limitations, the current report addresses the paucity of data in the literature for the burden of pneumonia in older adults in Australia.

Continued epidemiologic study of pneumococcal pneumonia in adults aged 65 years and older may serve to increase clinical awareness of disease burden.

## Conclusions

Pneumococcal disease in older adults in Australia represents a high clinical and economic healthcare burden. However, the burden of pneumonia remains less recognised than other diseases, such as influenza. The greatest burden of disease rests with pneumococcal pneumonia rather than IPD. Continued surveillance of pneumococcal pneumonia in adults living in Australia who are older than 65 years of age is essential.
